# A Mixed-Methods Approach to the Development, Refinement, and Pilot Testing of Social Networks for Improving Healthy Behaviors

**DOI:** 10.2196/humanfactors.4512

**Published:** 2016-02-12

**Authors:** Sarah Hales, Gabrielle Turner-McGrievy, Arjang Fahim, Andrew Freix, Sara Wilcox, Rachel E Davis, Michael Huhns, Homayoun Valafar

**Affiliations:** ^1^ Arnold School of Public Health Department of Health Promotion, Education, and Behavior University of South Carolina Columbia, SC United States; ^2^ Department of Computer Science University of South Carolina Columbia, SC United States; ^3^ Arnold School of Public Health Department of Exercise Science University of South Carolina Columbia, SC United States

**Keywords:** mHealth, obesity, weight loss, social support, social cognitive theory

## Abstract

**Background:**

Mobile health (mHealth) has shown promise as a way to deliver weight loss interventions, including connecting users for social support.

**Objective:**

To develop, refine, and pilot test the Social Pounds Off Digitally (POD) Android app for personalized health monitoring and interaction.

**Methods:**

Adults who were overweight and obese with Android smartphones (BMI 25-49.9 kg/m^2^; N=9) were recruited for a 2-month weight loss pilot intervention and iterative usability testing of the Social POD app. The app prompted participants via notification to track daily weight, diet, and physical activity behaviors. Participants received the content of the behavioral weight loss intervention via podcast. In order to re-engage infrequent users (did not use the app within the previous 48 hours), the app prompted frequent users to select 1 of 3 messages to send to infrequent users targeting the behavioral theory constructs social support, self-efficacy, or negative outcome expectations. Body weight, dietary intake (2 24-hr recalls), and reported calories expended during physical activity were assessed at baseline and 2 months. All participants attended 1 of 2 focus groups to provide feedback on use of the app.

**Results:**

Participants lost a mean of 0.94 kg (SD 2.22, *P*=.24) and consumed significantly fewer kcals postintervention (1570 kcal/day, SD 508) as compared to baseline (2384 kcal/day, SD 993, *P*=.01). Participants reported expending a mean of 171 kcal/day (SD 153) during intentional physical activity following the intervention as compared to 138 kcal/day (SD 139) at baseline, yet this was not a statistically significant difference (*P*=.57). There was not a statistically significant correlation found between total app entries and percent weight loss over the course of the intervention (*r=*.49, *P*=.19). Mean number of app entries was 77.2 (SD 73.8) per person with a range of 0 to 219. Messages targeting social support were selected most often (32/68, 47%), followed by self-efficacy (28/68, 41%), and negative outcome expectations (8/68, 12%). Themes from the focus groups included functionality issues, revisions to the messaging system, and the addition of a point system with rewards for achieving goals.

**Conclusions:**

The Social POD app provides an innovative way to re-engage infrequent users by encouraging frequent users to provide social support. Although more time is needed for development, this mHealth intervention can be disseminated broadly for many years and to many individuals without the need for additional intensive in-person hours.

## Introduction

Rates of overweight and obese US adults remain high with 69% having a body mass index (BMI) greater than 25 kg/m^2^ [1]. Obesity and being overweight are associated with type 2 diabetes, cardiovascular disease, arthritis, asthma [2,3], and some cancers—thyroid [4], colon, breast (in postmenopausal women), endometrium, esophagus, and kidney [5]. Behavioral interventions that target improvements in diet and physical activity are effective ways to help people lose weight and decrease chronic disease risk factors [6].

The use of apps on mobile devices (eg, smartphones and tablets) has the potential to improve how individuals monitor health behaviors by serving as a convenient platform to connect users to one another via online social networks. Mobile health (mHealth) holds promise as an effective method of delivering behavioral interventions addressing diet and physical activity, and it is less time-intensive than in-person, individual, or group sessions [7]. Mobile phone ownership is pervasive; 85% of US adults report owning a mobile phone with half owning smartphones [8]. Smartphone ownership cuts across ethnic groups with 49% of Hispanics, 47% of African Americans, and 42% of whites owning smartphones [8]. While there has been emerging research in the area of using mHealth technologies to help people achieve a healthy weight, few studies have used an entirely mobile device-based approach to deliver a behavioral weight loss intervention. Furthermore, many of the mobile-based weight loss apps available (both free and paid) do not include the evidence-based techniques used in traditional (ie, clinic-based) weight loss interventions [9,10].

Weight loss programs have been developed and delivered via Internet and other Web-based platforms as well as through social media (eg, Facebook [11] and Twitter [12]) to promote weight loss and reduce health risks of chronic disease. Frequent participant engagement with social media in the context of these weight loss interventions has been shown to be related to weight loss [11-13]. While there are many benefits to delivering weight loss interventions using remote methodologies, maintaining participant engagement over time and providing sufficient social support in these types of interventions can be a challenge [14].

The overarching objective of this line of research has been to design an app that can be used to monitor and test scientific hypotheses related to optimal matching of participants to provide support for collective weight loss in the context of mobile interventions. The primary goal for this pilot study was to solicit participant feedback to refine the Social Pounds Off Digitally (Social POD) app for use in a larger pilot randomized clinical trial (RCT). The Social POD app was developed by a transdisciplinary team of researchers including experts in health behavior, nutrition, computer science, psychology, exercise science, and social work. The analysis sought to answer the following questions: (1) What features of the Social POD app needed to be refined or developed to further incentivize participants to use the app? (2) Were there any significant changes from pre- to poststudy in participant weight, calories consumed, and reported intentional physical activity? (3) Was participant weight loss correlated with frequency of app use over the course of the study?

## Methods

### Participant Recruitment

Men and women who were overweight or obese with Android smartphones (BMI 25-49.9 kg/m^2^; N=9) were recruited in South Carolina for a 2-month weight loss pilot intervention to test usability and provide feedback to be used in the refinement of the smartphone app. Participants were recruited via worksite listserv announcements, flyers, and newspaper advertisements. Exclusion criteria included not having an Android phone, previous stroke or heart attack diagnosis, diagnosis of diabetes and using insulin or oral medications to control diabetes, BMI outside the range of 25.0-49.9 kg/m^2^, unable to attend required meetings, unable to access the Internet using a computer for completing assessments, having a psychiatric illness, receiving treatment for drug or alcohol dependency, having an eating disorder, participating in another weight loss program, being pregnant or planning on becoming pregnant during the study, and breastfeeding. Participants were excluded for endorsing any of the first 4 items on the revised Physical Activity Readiness Questionnaire (PAR-Q) [15,16]: (1) informed by a doctor that they have a heart condition and should only participate in physical activities approved by a doctor, (2) feeling chest pain when engaging in physical activity, (3) experiencing chest pain in the past month when not engaging in physical activity, and (4) ever losing their balance and becoming dizzy or ever losing consciousness. If participants reported a bone or joint problem that could be made worse by participating in physical activity (item 5 of the PAR-Q) or taking blood pressure medication (item 6 of the PAR-Q), they were required to have a physician consent form completed to participate in the study. Participants received US $30 for completion of assessments at baseline, US $15 after the 1-month focus group, and US $15 after the 2-month follow-up weight assessment.

### Intervention Implementation

Participants attended 4 in-person meetings: an orientation session to learn about the study and complete baseline dietary assessments; a training session to learn how to download and use the Social POD app and podcasts and to collect baseline height and weight measurements; a mid-study focus group at 1 month to provide feedback regarding the usability of the Social POD app, provide suggestions for improvement, and collect 1-month weight measurements; and an end-of-study session to provide 2-month weight measurements. All participants provided written consent. This study was approved by the University of South Carolina institutional review board.

**Figure 1:**
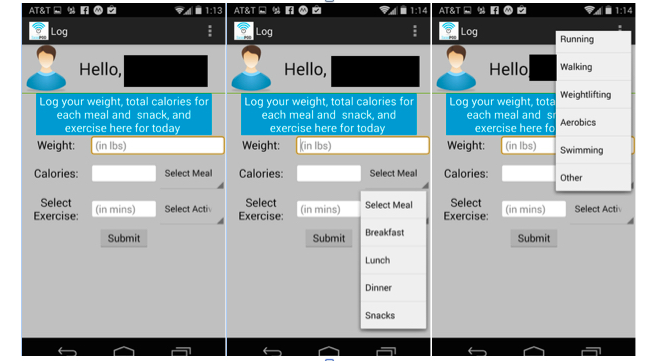
Screenshots (left to right) of the home, meal tracking, and physical activity tracking screens on the Social POD app.

Participants were instructed to track their total calories from all meals, snacks, and beverages consumed; minutes of intentional physical activity; and body weight each day for the duration of the 2-month usability study. Participants were instructed to use MyFitnessPal or LoseIt, free commercial diet-tracking apps with extensive nutrient databases, to look up calorie information for food and beverages consumed. Participants were then asked to transfer total calories from each meal and snack consumed for the day to the Social POD app. Screenshots of the tracking features are shown in Figure 1.

**Figure 2:**
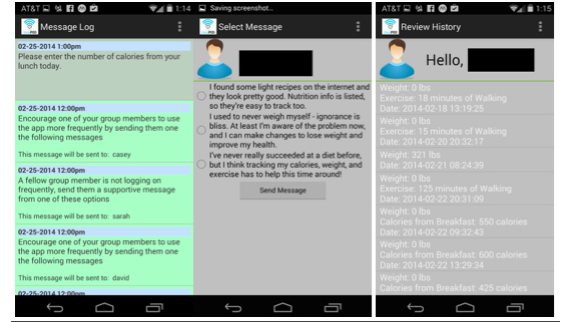
Screenshots (left to right) of the message log, message selection, and history screens on the Social POD app.

Participants received within-app notifications at certain times throughout the day to remind them to self-monitor (promoting self-regulation) diet, minutes of physical activity, and total body weight each day. Participants could view a history of all dietary, activity, and weight information entered on the within-app history screen. Participants could view weight entered on a weight graph. Participants who were frequently using the Social POD app (users who entered information in the app in the past 48 hours) were prompted via notifications to send encouraging messages to other group members who had not entered data in the app over the previous 48 hours (infrequent users). Messages were sent from frequent users by clicking the notification to send a message to an infrequent user, selecting one of three options listed on the message selection screen, and clicking Send. Screenshots of the message log, message selection, and history screen of the Social POD app features are shown in Figure 2.

This study is novel in that it used theory-based messages designed by the researchers to help re-engage infrequent participants over the course of the 2-month intervention. The participants were matched to provide support to one another based on principles of recommender systems used by some websites and applications (eg, Amazon and Netflix) [17-20], which filter information to match users based on user history or preferences [21]. Frequent users were randomly matched to provide support (by sending a theory-based message) to help re-engage infrequent users in this intervention. Social Cognitive Theory (SCT) [22,23] was used as a framework to design user-to-user messages that targeted social support [13], self-efficacy [22,23], and outcome expectations [24] regarding self-monitoring behavior (ie, targeting self-regulation) [23] of diet, physical activity, and weight. SCT is the belief in the reciprocal relationship between cognitions, environmental influences, and behavior [22,25]. Constructs from SCT were selected for this study based on results from a previous Internet-based weight loss intervention, which found that targeting these specific constructs led to healthier diet and physical activity behaviors and resulted in a reduction of body weight among study participants [26]. The interventionists created messages for each of the three social construct theories and prompted frequent users to select 1 of 3 messages to send to an infrequent user. Table 1 provides examples of each message type as well as the SCT construct targeted.

**Table 1:** User-to-user message types by social cognitive theory construct targeted.

SCT construct targeted	Construct definition	Sample message
Self-efficacy	One’s belief in the ability to perform specific tasks and overcome barriers.	“I found some light recipes on the Internet and they look pretty good. Nutrition info is listed, so they’re easy to track too.”
Social support	Support from others, which can take many forms including information, suggestions, or advice.	“Haven’t seen you log anything in the app lately. We miss you!”
Outcome expectations	Expected outcomes of behaviors.	“I’ve never really succeeded at a diet before, but I think tracking my calories, weight, and exercise has to help this time around!”

Participants were provided with 3 20-minute, evidence-based weight loss podcasts each week. Podcasts were uploaded to the study website, and participants were sent a reminder that a new podcast was available every Monday, Wednesday, and Friday via email. Participants were instructed to listen to the 3 podcasts within the week but could listen at a time and place of their choosing. Podcasts were informed by SCT and provided participants with a range of weight loss topics. Podcast topics included nutrition and exercise information, an audio diary tracking the weight loss progress with challenges experienced by a male and female character, and a weight loss soap opera depicting the challenges of overcoming social barriers to weight loss, with a goal-setting activity related to weight loss at the end of each episode. Specific information regarding the development and testing of these podcasts in previous interventions can be found elsewhere [12,27].

### Outcome Measures

Participants completed online questionnaires assessing demographic characteristics. Height was measured at baseline assessment only using a Seca 213 (Hamburg, Germany) calibrated stadiometer. Weight was measured at baseline and 2 months using a Seca 869 calibrated digital scale accurate to 0.01 kg. Dietary measures were completed by participants using the National Cancer Institute’s Automated Self-Administered 24-Hour Dietary Recall (ASA24) [28]. Each participant completed the ASA24 online for 1 unannounced weekday and weekend day at baseline and 2 months. The following previously validated questionnaires were completed by participants at baseline and 2 months unless otherwise specified: the Paffenbarger Physical Activity Questionnaire to determine calories expended during activity [29], the 20-item Weight Efficacy-Lifestyle Questionnaire to measure participant self-efficacy [30], the 44-item Big Five Inventory (baseline only) as a measure of personality characteristics [31], and the Sallis Social Support Scale [32] and Physical Activity Social Influence Scale as measures of social support from family and friends (modified to include online social networks) [33]. Objective measures of frequency and duration of app use were collected unobtrusively by a secure network server following the intervention (at 2 months). All baseline measurements were collected prior to the administration of the weight loss intervention.

Participants attended 1 of 2 focus group sessions at 1 month to provide feedback regarding their use of the intervention components (Social POD app and podcasts). The focus groups were conducted by a trained focus group facilitator, and questions were asked using a semistructured interview guide developed by the investigators that included prescripted and spontaneous probes designed to elicit information from participants regarding their experience with the Social POD app. Questions were designed to assess participant satisfaction and dissatisfaction with the weight loss podcasts; the Social POD app including the tracking, prompting, and messaging features; and the weight graph and history features for future revisions. Participants were also asked if they would like incentives for using the app, and if so, how they would like an incentive system to be structured. Information regarding aesthetics and appearance of the app was solicited as well.

Focus group sessions were taped using an audio recorder, and all participants were instructed to use their study identification number to protect confidentiality during the focus groups. A trained graduate research assistant was present at the focus group sessions to take field notes during the sessions. The focus group facilitator completed detailed memos following each midstudy focus group session to document salient themes from participant discussions. Recordings from the 1-month focus group were transcribed verbatim by the research assistant and were cross-referenced with recordings for accuracy.

During the study (weeks 1 through 7) participants completed brief weekly online surveys each Friday to report the number of podcasts listened to and the number of days they used the Social POD app that week. Participants also reported any problems experienced using the Social POD app during the past 7 days and any suggestions they had to improve the app. If participants reported problems or suggestions for the Social POD app, they were then asked to write a detailed explanation.

### Statistical Analysis

All qualitative data were collected and analyzed between February and August 2014 using NVivo for Mac Beta 2014 statistical software (QSR International) to extract a combination of a priori and emergent themes from the focus groups to guide the revision of the Social POD app. Open-ended responses from weekly surveys regarding problems experienced and suggestions for improvements to the Social POD app were coded as well. Stata version 13.1 (StataCorp) software was used to detect statistical significance for all quantitative data analysis. Paired samples *t* test was used to test for statistically significant differences from pre- to posttest for weight, calories consumed, and calories expended during reported physical activity. The effect size for weight loss was also calculated. Correlation between total app entries and percent weight loss at 2 months was examined. Correlation between total days of objectively measured app use and total days of self-reported app use (via weekly questionnaires) by participants was examined. Fisher exact test was used, due to the small sample size, to detect significant differences in user-to-user message selection among participants categorized into subgroups based on amount of weight loss (dichotomized into groups of high versus low weight loss at the median split of percent weight loss). The sample size for this study was determined for qualitative analysis based on expert recommendations for mHealth usability testing studies [34,35]; therefore, this study was not powered to detect significant changes in within-group weight. Changes in weight and weight-related behaviors, however, are presented.

## Results

### Demographic Characteristics

A total of 34 individuals inquired about the study, 15 individuals qualified to participate, and 9 completed baseline measures and received the intervention. Major reasons why participants did not qualify included not having an Android phone (9/19, 47%) and meeting medical exclusion criteria (3/19, 16%). Most participants (7/9, 78%) attended the 1-month focus group sessions (2 infrequent app users did not attend), and all participants completed the poststudy questionnaire and unannounced dietary recalls and had their body weight measured poststudy. The response rate for weekly surveys was 62% (39/63). Baseline demographics of the study sample are presented in Table 2.

**Table 2:** Baseline demographic characteristics.

Characteristics		n=9
Age (years), mean (SD)		39 (14.5)
**Gender, n (%)**		
	Female	8 (89)
	Male	1 (11)
**Race, n (%)**		
	Black	3 (33)
	White	6 (67)
Weight (kg), mean (SD)		91.5 (19.1)
Body mass index, mean (SD)		35.5 (7.1)
**Educational attainment, n (%)**		
	Advanced	3 (33)
	College	5 (56)
	Some college	1 (11)
**Marital status, n (%)**		
	Divorced or separated	1 (11)
	Married	2 (22)
	Single	4 (44)
	Other	2 (22)

### Focus Group Themes

Major themes that emerged during focus group discussions are reported below with illustrative quotes from participants. These themes included Social POD app functionality, improvements to the Social POD app and podcasts, additions to the Social POD app, incentive and goal system for the Social POD app, and satisfaction with the Social POD app and podcasts.

#### Functionality of Social POD App

Functionality problems reported by participants included issues with the notification system that prompted participants to track their daily diet, weight, and minutes of physical activity. Reported functionality issues with the notification system mentioned during the focus groups as well as on the weekly surveys included 1 failure to receive notifications and 1 failure of notifications to connect with the correct screen within the Social POD app. The same participant reported 2 instances of spontaneous crashing of the Social POD app on the weekly surveys.

#### Improvements to Social POD App and Podcasts

Themes relating to suggested improvements for the Social POD app were varied and included adding additional features and modifying existing features. Modifying the color scheme and integrating options for personalization were suggested. One participant suggested using colors that “get your attention more” by replacing the grey and dark blue used in the current version of the Social POD app with brighter colors. Another participant mentioned she would like to have had the opportunity to choose an avatar or icon for her home screen to customize the appearance of her app. Having the ability to track calories consumed on previous dates was another requested modification. Most participants (3 out of 4 participants in this group) reported that the notifications to track their diet, physical activity, and weight were helpful as reminders to track their healthy behaviors, although 1 participant said she found them to be annoying.

There were not many suggested improvements for the podcasts during the focus groups, but participants did note problems with sound quality and variation in the volume level. A participant in the focus groups mentioned that there was a difference in the volume level of the various segments of the podcasts, specifically with the soap opera portion. Participants recommended leveling the volume and improving the sound quality of the podcast episodes prior to future use. While all participants reported that they valued the information provided in the podcasts and felt satisfied with the various segments, some participants (n=3) reported they did not like the soap opera portion of the podcast episodes. A participant shared, “I don’t particularly like the soap opera,” and 2 members of the group agreed with this statement and said this story line was “geared toward young dating women” and “negative.” Another participant would have enjoyed the soap opera portion of the podcast more if all of the episodes could be listened to consecutively rather than having to wait until the next episode was available to download.

#### Additions to the Social POD App

Suggested additions to the Social POD app included adding a database of common foods and beverages within the app (versus having to use another app to look up calorie information) and incorporating an incentive system. Increasing the amount of praise provided by the study staff for entering weight and achieving weight loss–related goals was a highly suggested addition to the app. All of the participants that attended the focus groups (n=7) expressed their desire to see how others were doing in the program and to send other participants encouragement for achieving weight loss goals. A participant mentioned it would be interesting to see if messages sent to other users actually motivated them to use the app more often.

I think it’d be interesting to hear somebody from the receiving end to see if that motivated them to do something.

#### Incentive and Goal System of the Social POD App

All participants in the focus groups reported they would like an incentive system with rewards for using the tracking components of the Social POD app. Participants suggested that points should be redeemed for prizes.

I’m all about a token economy, so yes,...I think like a five year old, so yeah, that [prizes] really does motivate me.

Participants recommended earning points specifically for completing physical activity.

I haven’t been nearly as active as I would have liked to have been...having a point reward system for that would be an incentive for me to move more.

A participant mentioned that creating a competition among study participants or giving prizes for personal bests would be motivating.

I certainly like prizes [laughter]. It could also be fun to have a competition between the Social POD users so you could have the leader in points receive some prize or even personal bests, like if you have a 5-week program and your best week you could get some rewards.

Participants recommended the integration of preset or tailored goals to limit the possibility that some might set unrealistic goals for themselves and give up on the program.

First participant: Think of the other side if you decide for yourself some goals, and for some reason you cannot make them, how would you feel?

Second participant: Really terrible, but if it was somehow like...If the system could somehow help you set reasonable goals instead of like high in the sky things that I know I’m not going to do. People do tend to set lofty goals for themselves when really it should be small, measurable, incremental changes at first.

#### Satisfaction With Social POD App and Podcasts

Themes relating to satisfaction with Social POD app components included the user-to-user messaging system. Some participants preferred the prewritten user-to-user messages because they seemed more professional than allowing participants to create their own user-to-user messages.

I think the prepopulated messages is [are] the best because it’s professional.

Overall, participants reported satisfaction with the messaging system. A participant reported that she would have been less likely to send messages to others if she had to write the messages herself.

I know that I would be less likely to send a message to someone if I had to write it myself...so having some [messages] to choose from where it takes just a second to do, I’m more likely to do that.

Overall, participants reported the Social POD app was simple and easy to use. Participants reported they liked the convenience and ability to use their smartphones to track their diet, which they found to be more inconspicuous than other methods (eg, using a calorie book).

Every now and then you feel like James Bond because if you are at the table an[d] you still have your cellphone with you an[d] you turn on the app and...nobody knows what you do...an[d] you just...key in everything an[d] done. I mean [at] the end of the day...I’ve done something for myself today.

Participants also mentioned that the app gave them the motivation they needed to change their diet and physical activity behaviors to lose weight.

It’s definitely been a useful exercise...my thing is process, not perfection...in my case I’m doing a lot more than I was doing before.

All participants in the focus groups volunteered that they valued the information provided in the podcasts.

I liked the information...it reinforces what I’m sure most of us already know.

Participants reported a variety of methods used to listen to the weekly podcasts. A participant created an icon on the phone to easily access the podcasts and even set reminders to listen to the podcasts throughout the week.

I’ve set up the podcasts as an icon on my phone. So then I just go to the website and can play it from there, and then I have an alarm set up for Monday, Wednesday, and Friday because otherwise I’ll forget to [listen] even with an email I’ll probably forget.

Another participant reported listening to the podcasts from a laptop.

It’s easy, I told you, I listen to this on my laptop, and once I get the email, I just click on the link on my media system in the laptop [and] just open up, and I can see everything there. I wish they could stay. I wished at the end of the study [the podcasts] would not go away.

Participants reported listening to the podcasts in a variety of settings. Some participants reported listening to the podcasts while in the car.

I like to listen to it in the car when I’m going to be in the car for twenty or thirty minutes.

I like to listen to them in the car or when I’m getting ready in the morning and that’s a good time to do it, so I appreciate that your assistant sends them early in the morning, so I have a good time, a whole day really to listen to it.

All focus group participants mentioned that they liked at least one of the various segments included in the podcast episodes.

I also really like the section where you listen to someone’s diary...that’s helpful.

Another participant liked the diversity of characters included in the podcasts.

I like there’s an array of people who share. Older gentlemen, the women, whoever you seem to identify with you can find somebody who is good for you.

#### Suggested Improvements

On the weekly surveys, participants suggested many of the same improvements discussed in the focus group sessions. Suggestions for improvements to the Social POD app included adding more vibrant colors to the app, adding customization such as a personal avatar, integrating a nutrient database, adding the ability to track diet and activity for previous dates, and providing more praise for losing weight and/or increasing physical activity. Additional suggestions made on the weekly surveys not mentioned in the focus groups included adding additional activity options for tracking physical activity (n=1), giving participants the ability to customize the user-to-user messages (n=1), and revising the user-to-user messages to sound more motivating and encouraging (n=1).

### Planned Revisions of the Social POD App and Podcasts Based on Focus Group Themes

A news feed will be developed for the Social POD app for participants to view the progress of other users with weight loss-related goals. Participants will be able to send others encouragement for achieving these goals (eg, logging 30 minutes of exercise, logging diet and weight) through the news feed, targeting positive reinforcement [22]. Other revisions to the next iteration of the Social POD app will include the ability to earn points on a Point Tracker within the app for self-monitoring diet, physical activity, and weight and for sending others encouragement through the news feed. Points will be redeemed for study-provided prizes at the end of the pilot RCT, targeting reinforcement [22]. A weight loss competition among participants, as suggested by one participant, will not be integrated into the revised version of the Social POD app to minimize the potential risk of harm to some participants who are not achieving weight loss goals as quickly as others. Integrating a database of food and beverages similar to commercial diet tracking apps to view the nutrient content of commonly consumed items was recommended and will be incorporated into the calorie tracking features of the Social POD app to facilitate self-regulation [23]. A more extensive list of activities will be incorporated to the physical activity tracker in the revised Social POD app, also promoting self-regulation [23]. The color scheme of the app will be modified to include colors that are brighter and more eye-catching, and the option to add an avatar on the home screen will be incorporated. User-to-user messages will be revised to include more encouraging statements to better re-engage infrequent users with the Social POD app. Planned revisions for the podcasts include rerecording the podcast episodes to improve the sound quality and volume across segments within the episodes.

### Quantitative Results

Participants lost a mean of 0.94 kg (SD 2.22). Differences in mean participant weight before (91.48 kg, SD 19.08; 95% CI 76.82-106.15) and after (90.55 kg, SD 20.01; 95% CI 75.17-105.93) the 2-month intervention were not statistically significant (*P*=.24, *d=*.05, *r=*.02). Participants reported expending a mean of 171 kcal/day (SD 153) during intentional physical activity following the intervention as compared to 138 kcal/day (SD 139) at baseline, yet this was not a statistically significant difference (*P*=.57). Participants reported consuming significantly fewer calories following the intervention (1570 kcal/day, SD 508) than before (2384 kcal/day, SD 993, *P*=.01).

Mean number of Social POD app entries over the course of the 2-month usability study was 77.2 (SD 73.8, 95% CI 17.66-133.68) with a minimum of 0 and a maximum of 219 entries. Messages were sent by frequent users targeting social support most often (32/68, 47%), followed by self-efficacy (28/68, 41%) and outcome expectations (8/68, 12%). There was not a statistically significant correlation between total app entries and percent weight loss over the course of the intervention (*r=*.49, *P*=.19). There was no difference in the type of message selected (self-efficacy, social support, and outcome expectation) between those participants who were successful at weight loss as compared to those who were less successful (defined at median split in percent weight loss) (*P=*.79).

On the weekly surveys, participants reported listening to an average of 2.24 podcast episodes (SD 1.50; minimum 0, maximum 6) per week, and they reported using the Social POD app an average of 4.5 days (SD 2.25; minimum 0, maximum 7). There were 7 reports of problems using the Social POD app by 3 participants over the course of the 2-month study. Reported problems from weekly surveys included spontaneous crashing (2 times) and notifications linking with the incorrect screen in the Social POD app (1 time); there were no explanations for the other reported problems on the weekly surveys.

There were 2 participants with no objective measure of Social POD app use over the course of the study. A participant reported using the Social POD app 1 day during the study, and she specified on the weekly survey that she did not use the app because she did not have enough time. The other participant without objective app use data reported on the weekly surveys that she used the app a total of 21 days during the study. The total number of days participants used the app (as objectively measured by the app) and total number of days of self-reported app use by participants via weekly surveys was highly correlated (*r*=.87, *P*<.01), indicating that self-report was a reliable measure of app engagement in this study.

## Discussion

### Comparison to Prior Work

While there has been much work in the area of mobile app development for health, there is currently little published research in the area of development and testing of new mobile apps for weight loss among adults who are overweight and obese. There are several recent studies documenting the development and testing of new apps for weight loss in adolescents [36], apps for increasing physical activity and reducing screen time among adolescent males [37], and apps for predicting the risk of childhood obesity among infants [38]. Several recent studies report on the development and testing of apps for modification of diet and physical activity among the young adult population [39-41].

In a similar mixed-methods usability study, Morrison and colleagues used a computer-based weight loss program in conjunction with an Android mobile app to improve participant goal setting and motivation to achieve weight management goals among young adult participants over a 4-week period [39]. This app also offered participants the opportunity to set notifications, similar to those in the Social POD app, as reminders to use the diet and physical activity diary features. Participants could choose when and if they would like to receive notifications in an effort to improve the usability of and satisfaction with the mobile app [39]. It was found that only about half of the participants used the self-monitoring features of the app over the course of this usability testing [39]. While 1 participant in the Social POD app study noted that the notifications could be “annoying,” others found the notifications, which were preset by study coordinators, a helpful reminder to self-monitor their behaviors and weight and said that they otherwise would not have performed this task. Prior research has also demonstrated that weight loss is improved when self-monitoring activities are performed in real-time and proximal to the target behavior [42]. This indicates that mHealth apps could better help users self-monitor health-related behaviors using a notification system that cannot be eliminated by participants to remind them throughout the day and at times in which the behaviors typically occur.

In a qualitative study examining the desired features of weight loss apps among young adults, Tang and colleagues found that participants valued the opportunity to move beyond strictly tracking their eating behaviors and wanted a way to integrate other features, such as behavioral weight loss goals [40]. Other mobile apps have also incorporated a goals feature. Morrison and colleagues instructed participants to set their own goals and track their goal achievement progress using their mobile app and found that participants most frequently accessed informational content using their app (eg, food lists); fewer participants used the goal setting and monitoring features of their mobile app [39]. As some participants are less likely to use app components that require initial set-up (eg, setting goals or notifications), including some type of preset behavioral goals for users to achieve could help promote user engagement and motivation with these mHealth apps.

In their qualitative study documenting the development and prototype testing of an app promoting change in eating and activity behaviors to reduce weight gain among young adults, Hebden and colleagues received feedback requesting positive reinforcement for performing desired behaviors (eg, eating healthy foods or engaging in physical activity) [41]. This is similar to participant suggestions for more opportunities to provide and receive praise in the Social POD study. Including opportunities for users to give and receive praise for performing targeted health behaviors could be another necessary component to help establish and maintain health behavior change within the context of mHealth interventions.

The color scheme of the Social POD app will be updated with brighter colors to better appeal to users as suggested in the Social POD participant focus groups. Tang and colleagues conducted focus groups with young adult participants and also found that the perceived attractiveness of an app was an important consideration for participant satisfaction and maintaining engagement with weight loss apps [40]. Functionality issues similar to those found in the notification system of the Social POD app were found in a study conducted by Morrison and colleagues where a participant reported not receiving notifications during testing of a new mobile app for goal setting and self-monitoring of diet and activity among a small sample of adults [39].

Ensuring that all components of mHealth apps are functioning properly over time is an integral part of the usability testing process and of great importance in remotely delivered behavioral health interventions. Following usability testing, it was imperative to prioritize the changes that were made to the Social POD app prior to the pilot RCT. Correcting functionality issues and developing a newsfeed and incentive systems took priority; an avatar to personalize the app was not incorporated, and, while a nutrient database was added, it was not as extensive as originally hoped.

### Limitations

Because the purpose of this study was to test the first iteration of the Social POD app prior to the pilot RCT, it was not adequately powered to detect statistically significant differences in pre-post scores. The time period of the study was limited and at 2 months may have been too short to detect significant differences in participant pre-post body weight. The Social POD app is currently only available for the Android operating system. Despite the fact that the Android phone is the most prevalent cell phone in the United States [43], the fact that other smartphones (eg, iPhone) were excluded may reduce the generalizability of the findings. Direct questioning (versus open-ended questioning) was used to solicit participant opinions regarding the addition of an incentive system, which could have resulted in bias. The sample size of this study was also very small, at just 9 participants and only 1 male, and was therefore not a representative sample of all potential users of the Social POD app. Because the sample size was small, message selection results could be skewed toward the message type that frequent participants preferred. Participants were instructed to use a commercial database to identify the caloric content of food and beverages consumed and use of this database could have contributed to the change in weight observed following this intervention.

### Strengths

One strength of this study was the use of a mixed-methods design, which included participant focus groups to obtain usability and functionality feedback and suggestions for improvements to the Social POD app prior to the pilot RCT. Obtaining feedback through the weekly surveys was another strength, given the potential for some participants to refrain or modify comments during focus groups based on social desirability bias. Another strength of this study was the iterative testing of the Social POD app to uncover and resolve functionality issues prior to the pilot RCT [34]. While the total number of app entries was not statistically significantly correlated with percent weight loss, a correlation coefficient of *r*=.49 represents a fairly large effect size [44]. Furthermore, mean difference in calories expended during intentional physical activity from pre- to posttest was also not significantly different, but this represented a small effect and could be greater if tested among a larger sample and over a longer time period [44]. While the sample size was small in this usability study, the minimum percentage of problems identified during testing of mobile apps increased from 55% to 85%, respectively, when the sample size increased from 5 participants to 10 [45]. The reach of mHealth interventions such as this has the potential to be even greater than traditional face-to-face interventions, and even small changes in weight have the potential to impact public health outcomes and reduce disease risk [46]. A reduction in body weight as little as 1 kg, as seen in this study, has been associated with a 16% reduction in type 2 diabetes risk [47] demonstrating that this type of mHealth intervention is a scalable way to deliver a weight loss program with beneficial reduction of disease risk. The comparison of objective and subjective reports of app use is another strength of this study.

### Conclusion

The Social POD app provides an innovative way to encourage self-monitoring of dietary intake, weight, and physical activity while encouraging frequent users to provide social support to infrequent users. Although more time is needed for development, this mHealth intervention can be disseminated broadly for many years and to many individuals without the need for additional intensive in-person hours. The Social POD app should be tested in a larger clinical trial for a longer length of time to determine if changes in participant weight, calories consumed, and calories expended during physical activity are improved.

## Abbreviations

ASA24: Automated Self-Administered 24-hour Dietary Recall
BMI: body mass index
PAR-Q: Physical Activity Readiness Questionnaire
RCT: randomized clinical trial
Social POD: Social Pounds Off Digitally
SCT: social cognitive theory
